# Inter-hospital transfers and outcomes of critically ill patients with severe acute kidney injury: a multicenter cohort study

**DOI:** 10.1186/s13054-014-0513-1

**Published:** 2014-09-17

**Authors:** Paul Kudlow, Karen EA Burns, Neill KJ Adhikari, Benjamin Bell, David J Klein, Bin Xie, Jan O Friedrich, Ron Wald

**Affiliations:** Department of Psychiatry, University of Toronto, 250 College St., M5T 1R8 Toronto, ON Canada; Interdepartmental Division of Critical Care, University of Toronto, 200 Elizabeth St., M5G 2C4 Toronto, ON Canada; Department of Critical Care, St. Michael’s Hospital, 30 Bond St., M5B 1W8 Toronto, ON Canada; Li Ka Shing Knowledge Institute of St. Michael’s Hospital, 209 Victoria St., M5B 1W8 Toronto, ON Canada; Department of Critical Care Medicine and Sunnybrook Research Institute, Sunnybrook Health Sciences Centre, 2075 Bayview Ave, M4N 3M5 Toronto, ON Canada; Division of General Internal Medicine, University of Toronto, 200 Elizabeth St., M5G 2C4 Toronto, ON Canada; Departments of Obstetrics and Gynecology and Epidemiology and Biostatistics, Western University, 800 Commissioners Rd E, N6A 5W9 London, ON Canada; Division of Nephrology, St. Michael’s Hospital and University of Toronto, 30 Bond Street, Toronto, ON M5B 1W8 Canada

## Abstract

**Introduction:**

Patients with severe acute kidney injury (AKI) who are hospitalized at centers that do not provide renal replacement therapy (RRT) are frequently subjected to inter-hospital transfer for the provision of RRT. It is unclear whether such transfers are associated with worse patient outcomes as compared with the receipt of initial care in a center that provides RRT. This study examined the relationship between inter-hospital transfer and 30-day mortality among critically ill patients with AKI who received RRT.

**Methods:**

We conducted a retrospective cohort study of all critically ill patients who commenced RRT for AKI at two academic hospitals in Toronto, Canada. The exposure of interest was inter-hospital transfer for the administration of RRT. We evaluated the relationship between transfer status and 30-day mortality (primary outcome) and RRT dependence at 30 days following RRT initiation (secondary outcome), by using multivariate logistic regression with adjustment for patient demographics, clinical factors, biochemical indices, and severity of illness.

**Results:**

Of 370 patients who underwent RRT for AKI, 82 (22.2%) were transferred for this purpose from another hospital. Compared with non-transferred patients who started RRT, transferred patients were younger (61 ± 15 versus 65 ± 15 years, *P* = 0.03) and had a higher serum creatinine concentration at RRT initiation (474 ± 295 versus 365 ± 169 μmol/L, *P* = 0.002). Inter-hospital transfer was not associated with mortality (adjusted odds ratio 0.61, 95% confidence interval 0.33 to 1.12) or RRT-dependence (adjusted odds ratio 1.64, 95% confidence interval 0.70 to 3.81) at 30 days.

**Conclusions:**

Within the limitations of this observational study and the potential for residual confounding, inter-hospital transfer of critically ill patients with AKI was not associated with a higher risk of death or dialysis dependence 30 days after the initiation of acute RRT.

**Electronic supplementary material:**

The online version of this article (doi:10.1186/s13054-014-0513-1) contains supplementary material, which is available to authorized users.

## Introduction

Acute kidney injury (AKI) requiring renal replacement therapy (RRT) occurs in approximately 3% to 10% of patients admitted to the intensive care unit (ICU) and is associated with short-term mortality in excess of 50% [1,2]. Since some hospitals may not have RRT capability, patients with severe AKI often require transfer to centers that offer RRT. Transfers may delay initiation of RRT and this conceivably could adversely impact upon outcomes [3]. Several large-scale studies have evaluated the association between inter-hospital transfer and outcomes among diverse populations of critically patients [4-7]. It remains unclear whether delays in care and the transfer process itself are mediators of adverse outcomes or whether heightened mortality among transferred patients is merely the result of confounding by acute illness or chronic comorbidity or both. In the specific setting of severe AKI, in which the application of renal support may directly treat and reverse some of the metabolic complications of this condition, it is plausible that delays associated with transfer can lead to worse outcomes. At present, there are no data on the impact of inter-hospital transfer on outcomes of critically ill patients with AKI who received RRT.

We conducted a two-center retrospective cohort study to examine the relationship between inter-hospital transfer and 30-day mortality among critically ill patients with AKI who initiated RRT. We hypothesized that patients who were initially admitted to a facility that did not provide RRT and were subsequently transferred to another hospital for the provision of RRT would have higher mortality and a higher risk of RRT dependence compared with individuals with RRT requiring AKI who commenced their hospitalization at centers with on-site RRT capability.

## Materials and methods

### Study design and datasets

This was a retrospective cohort study that comprised all patients who commenced RRT for AKI in an ICU setting at two academic medical centers in Toronto, Canada: St. Michael’s Hospital (April 2007 to December 2010) and Sunnybrook Health Sciences Centre (January to December 2010). We excluded patients with pre-existing end-stage renal disease, patients dialyzed solely for toxin removal, and transferred patients who commenced RRT at another hospital. The research ethics boards at both St. Michael’s Hospital and Sunnybrook Health Science Centre approved the protocol and waived the need for written informed consent. Written informed consent was waived because the study was retrospective with no potential for harm to subjects. All information was collected in an anonymized database, thereby ensuring patient privacy during the analyses.

### Data collection

Data were collected in a standardized fashion by trained data collectors using a Microsoft Access 2003 (Microsoft Corporation, Redmond, WA, USA) database. Inter-hospital transfer status and demographic, clinical, physiologic, and laboratory details were collected on the day of initial hospital admission, transfer to study hospital (if relevant), ICU admission at the study hospital, and RRT initiation. The burden of pre-existing chronic illness was assessed with the Charlson comorbidity index [8]. Severity of acute illness was recorded by using the modified Sequential Organ Failure Assessment (SOFA) score [9] calculated on the day of RRT initiation (Additional file 1). Relevant laboratory data were recorded on admission to hospital, admission to the ICU, and the day of RRT initiation, where available. Although premorbid kidney function was evaluated by using the estimated glomerular filtration rate (eGFR) as derived from the abbreviated Modification of Diet in Renal Disease (MDRD) formula [10], significant data were missing. The serum creatinine (sCr) value used for this calculation was the pre-hospitalization value closest to the day of admission but no more than 1 year prior to admission. When a pre-hospitalization sCr value was not available, the first sCr measured on admission to the hospital served as the baseline sCr. We documented blood pressure and vasopressor requirements at the start of RRT. The initial RRT modality was categorized as intermittent hemodialysis (prescribed treatment duration of less than 6 hours), sustained low-efficiency dialysis (prescribed treatment duration of more than 6 hours), or continuous RRT.

### Definition of exposure: transfer status

We defined patients as “transferred” if their hospitalization commenced at a different center and RRT was initiated within 2 calendar days of arrival at the study hospital. We chose this cutoff in order to define a cohort of patients in whom AKI and specifically the associated need for RRT were the driving stimuli for patient transfer. Patients admitted directly to the study hospitals where RRT commenced were considered “non-transfers”. Patients whose hospitalization commenced at a different center and started RRT of more than 2 days after transfer were included in the “non-transfer” group for the primary analysis as it is likely that the need for RRT was not the primary reason for their transfer. We performed two pre-specified sensitivity analyses to test the appropriateness of this assumption by (a) analyzing patients who commenced RRT of more than 2 days after transfer as a separate (third) group and (b) excluding such patients completely.

### Outcomes

The primary outcome was all-cause mortality, and the secondary outcome was dialysis dependence, both evaluated at 30 days after RRT initiation. Renal recovery was further described as complete, partial, or absent at 30 days following acute RRT initiation. Absent recovery was defined as persistent dialysis dependence. Complete recovery was defined as a return of the sCr to within 27 μmol/L of the initial baseline and not exceeding 1.5 times this value. In cases in which no pre-hospitalization baseline sCr was unavailable, an eGFR of more than 60 mL/min per 1.73 m^2^ 30 days after RRT initiation was considered to be complete recovery. RRT-independent patients who did not meet criteria for complete recovery were classified as having a partial recovery. In cases in which no follow-up sCr value was measured at 30 days, the last measured sCr was carried forward for the purposes of defining renal recovery. Finally, in the absence of follow-up data, if a patient was discharged from the study with an ongoing need for RRT but prior to 30 days after RRT initiation, we assumed the patient was still RRT-dependent on day 30.

### Statistical analyses

Descriptive statistics were used to compare baseline characteristics, clinical variables, and outcomes at 30 days among transferred versus non-transferred patients. Normally distributed continuous variables are described as means ± standard deviations and were compared by using the Student *t* test. Variables that were not normally distributed were compared by using the Kruskal-Wallis test. Categorical variables were compared by using the chi-square or Fisher exact tests. Multivariable logistic regression was used to identify the independent association between transfer status and the outcomes of interest after adjustment for potential confounders of this relationship (that is, age, sex, ICU type, Charlson score, SOFA score, selected features of clinical history, and laboratory values). These variables were selected due to clinical relevance. A *P* value of less than 0.05 was considered statistically significant. We performed all analyses by using SAS software version 9.4 (SAS Institute Inc., Cary, NC, USA).

## Results

### Baseline characteristics of the cohort

**Figure 1:**
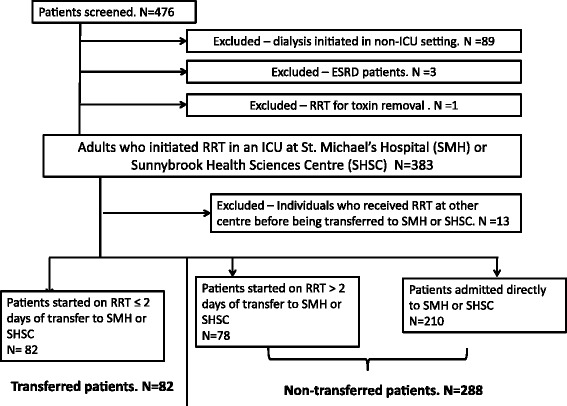
**Assembly of the study cohort.**

We identified 383 critically ill patients who received RRT for AKI during the study period (325 at St. Michael’s Hospital and 58 at Sunnybrook Health Sciences Centre). Thirteen patients were excluded as RRT was initiated at a different hospital prior to transfer to the study hospital. Of the remaining 370 patients, 82 (22.2%) were transferred and commenced RRT within 2 days of transfer, 78 were transferred but started RRT more than 2 days after transfer, and 210 were admitted directly to the study hospital at which RRT commenced (Figure 1). The 288 patients in the latter two groups were categorized as “non-transferred” as previously specified. Transferred patients were younger than non-transferred patients (61 ± 15 versus 65 ± 15 years, *P* = 0.03) and were more likely to have a medical diagnosis, to be managed in a general medical-surgical ICU, and to have worse kidney function on admission (Table 1). At the time of RRT initiation (Table 2), transferred patients had a significantly higher sCr (474 ± 295 versus 365 ± 169 μmol/L among non-transferred patients, *P* = 0.002) and a shorter interval from admission to RRT initiation (median 3, interquartile range (IQR) 1 to 7 versus 7, IQR 3 to 15 days among non-transferred patients, *P* <0.0001).

**Table 1:** **Patient characteristics by transfer status**

	**Transferred (n = 82)**	**Non-transferred (n = 288)**	***P*** **value**
Age, years	61 (15)	65 (15)	003
Male sex	49 (60)	187 (65)	0.39
sCr, μmol/L			
On admission	334.1 (358.9)	195.1 (187.0)	0.0013
At time of nephrology consult	459.3 (312.1)	291.9 (169.9)	<0.0001
On admission to the ICU	452.2 (325.8)	214.7 (185.5)	<0.0001
BUN, mmol/L			
On admission	19.1 (17.3)	14.6 (12.0)	0.04
On admission to the ICU	24.4 (14.6)	16.1 (12.4)	<0.0001
Diagnostic category			<0.001
Medical	55 (67)	113 (39)	
Surgical	27 (33)	175 (60)	
Cardiac surgery	5 (6.1)	65 (22.6)	0.0008
Abdominal aortic aneurysm repair	3 (3.7)	22 (7.6)	0.32
ICU type			0.002
Medical-surgical	66 (80.5)	179 (62.2)	
Coronary care	8 (9.8)	31 (10.8)	
Cardiovascular surgery	7 (8.5)	2 (0.7)	
Burn unit	1 (1.2)	76 (26.4)	
Charlson comorbidity index	2.7 (2.5)	2.7 (2.4)	0.78

Continuous variables are presented as means (standard deviation) unless otherwise specified and categorical variables as number (percentage). BUN, blood urea nitrogen; ICU, intensive care unit; sCr, serum creatinine.

**Table 2:** **Patient characteristics at initiation of renal replacement therapy**

	**Transferred (n = 82)**	**Non-transferred (n = 288)**	***P*** **value**
Median days from initial admission to RRT start at study hospital (interquartile range)	3 (1-7)	7 (3-15)	<0.0001
First RRT modality			
Intermittent hemodialysis	36 (43.9)	123 (42.7)	0.32
Continuous RRT	32 (39.0)	132 (45.8)	
Sustained low-efficiency dialysis	14 (17.1)	33 (11.5)	
Sequential Organ Failure Assessment score	14.4 (4.5)	14.2 (4.4)	0.70
Mechanical ventilation	64 (78)	229 (80)	0.77
Receipt of vasopressors	49 (60)	142 (49)	0.09
Systolic blood pressure, mm Hg	115 (22)	116 (23)	0.58
Serum creatinine, μmol/L	474 (295)	365 (169)	0.002
Urine output, mL/24 hours	396 (686)	479 (721)	0.35
Hemoglobin, g/L	91.0 (19.6)	87.0 (17.4)	0.07
White blood cell count, ×10^9^/L	14.3 (7.3)	15.8 (12.5)	0.17
Serum potassium, mmol/L	4.8 (1.0)	4.7 (0.9)	0.30
Serum bicarbonate, mmol/L	17.6 (5.7)	19.0 (5.4)	0.04
pH	7.27 (0.12)	7.30 (0.11)	0.05

Continuous variables are presented as means (standard deviation) or median (Q1-Q3) and categorical variables as number (percentage). pH data missing for three transferred and 37 non-transferred patients. RRT, renal replacement therapy.

### Outcomes

There was no statistically significant difference in crude 30-day mortality between transferred and non-transferred patients (42.7% versus 52.1%, respectively, *P* = 0.13). Among surviving patients, the likelihood of full kidney recovery was lower and the probability of persistent RRT dependence was higher among transferred patients (*P* = 0.02) (Table 3).

**Table 3:** **Unadjusted outcomes at 30 days following initiation of renal replacement therapy initiation**

**Outcomes**	**Transferred (n = 82)**	**Non-transferred (n = 288)**	***P*** **value**
Mortality, n (%)	35 (42.7)	150 (52.1)	0.13
Kidney status among survivors, n (%)			
Full recovery	6 (12.8)	46 (33.3)	0.02
Partial recovery	20 (42.6)	42 (30.4)	
Renal replacement therapy-dependent	21 (44.7)	50 (36.2)	

### Multivariable analyses

After confounders were accounted for, there was no association between inter-hospital transfer and 30-day mortality (adjusted odds ratio (OR) 0.61, 95% confidence interval (CI) 0.33 to 1.12 versus non-transferred patients). In the fully adjusted model, the only significant predictors of 30-day mortality were SOFA score (OR 1.19, 95% CI 1.09 to 1.31 per 1-point increase) and Charlson score (OR 1.15, 95% CI 1.03 to 1.28 per 1-point increase) at baseline (Table 4). Inter-hospital transfer was not independently associated with dialysis dependence at 30 days (adjusted OR 1.64, 95% CI 0.70 to 3.81) (Additional file 2). There were no independent significant predictors of dialysis dependence at 30 days.

**Table 4:** **The association between transfer status and 30-day mortality**

	**Univariate odds ratio (95% CI)**	***P*** **value**	**Multivariable odds ratio (95% CI)**	***P*** **value**
Transferred	0.69 (0.42-1.12)	0.13	0.61 (0.33-1.12)	0.11
Age, per year	1.00 (0.99-1.02)	0.48	1.02 (1.00-1.04)	0.02
Male	1.15 (0.75-1.76)	0.52	1.34 (0.79-2.25)	0.27
Surgical	0.88 (0.58-1.32)	0.53	0.91 (0.52-1.59)	0.73
Cardiac surgery	0.70 (0.42-1.19)	0.19	0.52 (0.26-1.03)	0.06
Post-AAA repair	0.54 (0.23-1.26)	0.15	0.47 (0.17-1.29)	0.14
Charlson score, per unit	1.08 (0.99-1.17)	0.11	1.15 (1.03-1.28)	0.01
**Variables at the start of renal replacement therapy**				
SOFA score, per unit	1.22 (1.16-1.29)	<0.0001	1.19 (1.09-1.31)	0.0001
Mechanical ventilation	2.50 (1.47-4.25)	0.0007	0.82 (0.40-1.69)	0.59
Vasopressors	3.41 (2.23-5.23)	<0.0001	1.25 (0.68-2.32)	0.47
Systolic blood pressure, per 10 mm Hg	0.79 (0.72-0.88)	<0.0001	0.87 (0.78-0.98)	0.03
sCr, per 50 μmol/L	0.86 (0.81-0.92)	<0.0001	0.91 (0.84-0.98)	0.01
Urine output, per 100 mL/day	0.99 (0.96-1.02)	0.36	1.01 (0.97-1.04)	0.69
Hemoglobin, per g/L	1.00 (0.98-1.01)	0.41	1.00 (0.99-1.01)	0.94
White blood cell count, per 1 × 10^9^ cells/L	1.00 (0.99-1.02)	0.75	0.99 (0.97-1.01)	0.39
Serum bicarbonate, per mmol/L	0.96 (0.92-1.00)	0.03	0.98 (0.94-1.03)	0.37

AAA, abdominal aortic aneurysm; CI, confidence interval; sCr, serum creatinine; SOFA, Sequential Organ Failure Assessment.

### Sensitivity analyses

We examined the influence of patients who commenced RRT more than 2 days after transfer (who were considered to be non-transferred in the primary analysis) on the robustness of our main results. We first analyzed these patients in their own category. After adjustment for confounders, transferred patients who commenced RRT within 2 days of transfer (adjusted OR 0.69, 95% CI 0.37 to 1.28) and those who commenced RRT more than 2 days after transfer (adjusted OR 1.82, 95% CI 0.96 to 3.46) did not have a higher adjusted 30-day mortality when compared with patients admitted directly to the study centers (“true” non-transferred patients) (Additional file 3). In a second sensitivity analysis, we excluded patients who commenced RRT of more than 2 days after transfer; the lack of association between inter-hospital transfer and 30-day mortality was consistent with the findings of the primary analysis (adjusted OR 0.68, 95% CI 0.36 to 1.28) (Additional file 4).

## Discussion

Critically ill patients who required inter-hospital transfer for the receipt of RRT, compared with patients who were admitted directly to the hospital at which RRT commenced, did not have a higher risk of 30-day mortality or RRT dependence. This is despite the fact that transferred patients commenced RRT with a higher sCr, suggesting that this group either had a higher prevalence of chronic kidney disease at baseline or initiated RRT when AKI was more advanced.

To the best of our knowledge, this is the first study to examine the impact of inter-hospital transfer on clinical outcomes among critically ill patients with AKI who received RRT. Our findings do not support the initial hypothesis that patients subjected to inter-hospital transfer in the context of severe AKI would have inferior outcomes compared with individuals who were admitted directly to a center at which RRT could be administered. Crude mortality was actually lower among patients who were transferred for RRT, an association that was attenuated to become statistically non-significant with adjustment for key confounders.

Inter-hospital transfers are associated with potential adverse events and the theoretical risk of clinical instability during transport [11,12]. These factors are compounded by data suggesting that delays in nephrology consultation and RRT initiation in hospitalized patients with AKI are associated with inferior clinical outcomes [3,13-16]. Although the controversy surrounding the timing of RRT initiation in AKI generally assumes that RRT is available on-site and that clinician decision-making is the main factor that determines timing of RRT initiation, the lack of RRT capability at many hospitals in Ontario presented a natural setting in which to study the effects of delayed initiation of RRT in the setting of AKI. Although a definitive trial in this area is needed, the current findings suggest that the ability to provide “urgent” RRT by either expanding local RRT capabilities or by accelerated transfer policies may not improve survival.

This is the first study to evaluate the impact of transferring patients with AKI and can be interpreted in the context of studies that examined the transfer of critically ill patients in other settings. A prospective cohort study [4] (n = 4,208) found that, compared with directly admitted patients, ICU patients transferred from another hospital had significantly higher acute physiology scores at the time of admission and discharge. Even after full adjustment for severity of illness, transferred patients had prolongation of ICU and hospital stays by 38% and 41%, respectively, and a greater than twofold higher risk of hospital mortality than directly admitted patients [4]. Increased mortality and length of stay among transferred patients have been described in other observational studies [5,6]. In contrast, a propensity-matched cohort study (n = 2,277) found that patients transferred between ICUs at two different hospitals for non-clinical reasons stayed on average 3 days longer in ICU compared with matched non-transferred patients, but there was no difference in mortality [7]. Overall, the impact of inter-hospital transfer on clinical outcomes is inconclusive and may depend on the context (that is, clinical versus logistic reasons for inter-hospital transfer).

This study has several strengths. Our cohort comprised critically ill patients from two urban tertiary care centers that frequently accept critically ill patients through a government-administered network that ensures the timely transfer of patients with specialized medical needs that cannot be met at the hospital to which they are admitted (for example, RRT). The study was set in a large province with many hospitals, some of which were remote from urban centers and do not provide RRT. As a result, our study addresses a challenge relevant to any health-care system providing care in a geographically large territory. We minimized bias by adjusting for several crucial confounders of the relationship between inter-hospital transfer and the outcomes of interest.

There are, however, important limitations to consider. Although there were no obvious differences between the transferred and non-transferred patients with respect to demographics or severity of illness, one must consider the very real possibility of residual confounding. For example, patients who underwent inter-hospital transfer may have been perceived as clinically healthier and thus able to withstand the transfer safely. As a result, the subset of individuals who were transferred may not reflect the population of patients at the sending hospital in whom transfer was not considered; had they been admitted initially to a hospital that provided RRT, they may have been offered RRT. This immortal time bias, as well as indication bias, may explain the trend toward improved survival among patients subjected to inter-hospital transfer. Secondly, our dataset does not provide information on the distance from the initial admitting hospital to the RRT-providing center and the travel time associated with the inter-hospital transfer. Some patients may have experienced more delays relative to others because of inter-hospital transfer, and we could not adequately capture this. The relatively small sample size may have also limited our statistical power to detect a significant association between transfer and clinical outcomes, and future studies with larger sample sizes will be needed to further clarify this question. Furthermore, we could not readily document the precise rationale for transfer in each case. As a result, we applied a somewhat arbitrary definition for the main exposure of “transfer status” that considered the patient to be transferred if he or she started RRT for AKI within two days of arriving at the RRT facility. We reasoned that if RRT was not started within two days of an inter-hospital transfer, the need for RRT was unlikely to be an important factor in the transfer of the patient. It is reassuring that our sensitivity analyses demonstrated that alternative ways of classifying this scenario did not affect the main results.

## Conclusions

Transfer of patients for the receipt of specialized medical care is an important public health issue with resource ramifications. Our findings suggest that patients with AKI who required inter-hospital transfer for the initiation of RRT did not have inferior clinical outcomes. Although this question would benefit from further study in larger cohorts and with consideration of AKI patients who are not transferred, the current data does not support the investment of resources to ensure the widespread establishment of RRT capability simply to mitigate the need for patient transfer.

## Key messages

- To the best of our knowledge, this is the first study to examine the impact of inter-hospital transfer on clinical outcomes among critically ill patients with AKI who received RRT.
- Within the limitations of this observational study and the potential for residual confounding, inter-hospital transfer of critically ill patients with AKI was not associated with a higher risk of death or dialysis dependence 30 days after the initiation of acute RRT.

## Additional files

Additional file 1:
**Modified Sequential Organ Failure Assessment (SOFA) score.** Patients were given a SOFA score (total 0 to 24). Additional file 2:
**The association between transfer status and dialysis-dependence at 30 days among surviving patients.**
Additional file 3:
**Sensitivity analysis.** The adjusted association between transfer status and 30-day mortality while considering patients who received renal replacement therapy (RRT) more than 2 days after transfer in a separate category is shown. Additional file 4:
**Sensitivity analysis.** The adjusted association between transfer status and 30-day mortality after excluding transferred patients who commenced renal replacement therapy (RRT) more than 2 days after transfer is shown.

## Abbreviations

AKI: acute kidney injury
CI: confidence interval
eGFR: estimated glomerular filtration rate
ICU: intensive care unit
IQR: interquartile range
OR: odds ratio
RRT: renal replacement therapy
sCr: serum creatinine
SOFA: Sequential Organ Failure Assessment
