# Supplementary material for: Inter-hospital transfers and outcomes of critically ill patients with severe acute kidney injury: a multicenter cohort study
Source: Crit Care. 2014 Sep 17;18(5):513. doi: 10.1186/s13054-014-0513-1 (PMC4189586; doi:10.1186/s13054-014-0513-1)
Supplement: Additional file 1: — Modified Sequential Organ Failure Assessment (SOFA) score. Patients were given a SOFA score (total 0 to 24). [file 13054_2014_513_MOESM1_ESM.docx]

**Additional file 1: MODIFIED Sequential Organ Failure Assessment (SOFA) SCORE**

**(Based on most extreme result for each category during the period of assessment.)**

| \| SOFA Score \| 0 \| 1 \| 2 \| 3 \| 4 \| \| --- \| --- \| --- \| --- \| --- \| --- \| \| ***Respiration***  **PaO_2_/FiO_2_** \| ≥ 400 \| ≤ 400 ≤ 300  (± resp. support) \| \| ≤ 200 ≤ 100  (+ resp. support) \| \| \| ***Coagulation***  **Platelets(x 10^9^/L)** \| ≥150 \| ≤ 150 \| ≤ 100 \| ≤ 50 \| ≤ 20 \| \| ***Liver***  **Bilirubin (µmol/L)** \| < 20 \| 20-32 \| 33-101 \| 102-204 \| > 204 \| \| **Cardiovascular** \| MAP ≥  70 mmHg \| MAP < 70 mmHg \| DA ≤ 5 µg/kg/min or dobutamine  or milrinone (any dose) \| DA > 5 µg/kg/min or  EPI ≤ 0.1 µg/kg/min or NE ≤0.1  µg/kg/min  or VP ≤ 0.02 U/min \| DA > 15 µg/kg/min or EPI > 0.1 µg/kg/min or NE > 0.1 µg/kg/min or VP ≥  0.03 U/min \| \| ***CNS***  **Glasgow Coma Scale (see below)** \| 15 \| 13-14 \| 10-12 \| 6-9 \| < 6 \| \| ***Renal****  **Creatinine (µmol /L)** \| < 110 \| 110-170 \| 171-299 \| 300-440 or urine output 200-499 mL/d \| ≥ 440 or urine output < 200 mL/d \| |
| --- | --- | --- | --- | --- | --- | --- | --- | --- | --- | --- | --- | --- | --- | --- | --- | --- | --- | --- | --- | --- | --- | --- | --- | --- | --- | --- | --- | --- | --- | --- | --- | --- | --- | --- | --- | --- | --- | --- | --- | --- | --- | --- |

**DA= dopamine EPI= epinephrine NE= norepinephrine VP = vasopressin**

*** If patient received any form of renal replacement therapy during the day of assessment, an automatic Renal score of 4 is assigned.**

**SOFA SCORE (total 0-24):** ______________
