# Supplementary material for: Inter-hospital transfers and outcomes of critically ill patients with severe acute kidney injury: a multicenter cohort study
Source: Crit Care. 2014 Sep 17;18(5):513. doi: 10.1186/s13054-014-0513-1 (PMC4189586; doi:10.1186/s13054-014-0513-1)
Supplement: Additional file 2: — The association between transfer status and dialysis-dependence at 30 days among surviving patients. [file 13054_2014_513_MOESM2_ESM.docx]

**Additional file 2: The association between transfer status and dialysis-dependence at 30 days among surviving patients**

|  | **Univariate Odds Ratio (95% CI)** | **p-value** | **Multivariable Odds Ratio (95%CI)** | **p-value** |
| --- | --- | --- | --- | --- |
| Transferred | 1.42 (0.73-2.78) | 0.30 | 1.64 (0.70-3.81) | 0.25 |
| Age, per year | 1.02 (0.99-1.04) | 0.15 | 1.01 (0.98-1.03) | 0.60 |
| Male | 0.99 (0.54-1.82) | 0.97 | 1.06 (0.51-2.20) | 0.88 |
| Surgical | 1.22 (0.67-2.21) | 0.53 | 1.18 (0.53-2.65) | 0.69 |
| Cardiac surgery | 1.25 (0.61-2.54) | 0.54 | 1.05 (0.41-2.68) | 0.92 |
| Post-AAA repair | 1.28 (0.45-3.59) | 0.64 | 1.25 (0.38-4.15) | 0.72 |
| Charlson score, per unit | 1.14 (0.99-1.32) | 0.07 | 1.10 (0.94-1.29) | 0.25 |
|  | Variables at the start of renal replacement therapy | | |  |
| SOFA score, per unit | 0.93 (0.86-1.00) | 0.04 | 0.92 (0.81-1.05) | 0.21 |
| Mechanical Ventilation | 0.80 (0.41-1.53) | 0.49 | 1.32 (0.55-3.19) | 0.54 |
| Vasopressors | 0.66 (0.36-1.24) | 0.20 | 0.90 (0.36-2.20) | 0.82 |
| Systolic blood pressure, per 10 mmHg | 1.08 (0.96-1.23) | 0.20 | 1.03 (0.88-1.20) | 0.71 |
| sCr, per 50 µmol/L | 1.03 (0.97-1.09) | 0.42 | 1.02 (0.94-1.11) | 0.60 |
| Urine output, per 100 mL/d | 0.90 (0.83-0.98) | 0.01 | 0.88 (0.80-0.97) | 0.009 |
| Hemoglobin, per g/L | 0.99 (0.98-1.01) | 0.50 | 0.99 (0.97-1.02) | 0.60 |
| White blood cell count, per 1 x 10^9^ cells/L | 0.98 (0.95-1.02) | 0.28 | 0.99 (0.97-1.02) | 0.53 |
| Serum bicarbonate, per mmol/L | 1.09 (1.03-1.15) | 0.004 | 1.08 (1.01-1.16) | 0.03 |

**^a^** Reference indicator variable.

**AAA=abdominal aortic aneurysm, SOFA=Sequential Organ Failure AssessmentsCr=serum creatinine**
