# Supplementary material for: Inter-hospital transfers and outcomes of critically ill patients with severe acute kidney injury: a multicenter cohort study
Source: Crit Care. 2014 Sep 17;18(5):513. doi: 10.1186/s13054-014-0513-1 (PMC4189586; doi:10.1186/s13054-014-0513-1)
Supplement: Additional file 3: — Sensitivity analysis. The adjusted association between transfer status and 30-day mortality while considering patients who received renal replacement therapy (RRT) more than 2 days after transfer in a separate category is shown. [file 13054_2014_513_MOESM3_ESM.docx]

**Additional file 3. Sensitivity analysis. The adjusted association between transfer status and 30-day mortality while considering patients who received RRT greater than 2 days after transfer in a separate category**

| **Variable** |  | **Univariate OR**  **(95% CI)** | **Multivariable OR (95% CI)** |
| --- | --- | --- | --- |
| Group | Non-transfer | 1.00 | 1.00 |
|  | Transferred and started on RRT ≤2days | 0.72 (0.43-1.20)  p=0.20 | 0.69 (0.37-1.28)  p=0.24 |
|  | Transferred and started on RRT >2days | 1.18 (0.70-1.99)  p=0.53 | 1.82 (0.96-3.43)  p=0.07 |
