# Supplementary material for: Inter-hospital transfers and outcomes of critically ill patients with severe acute kidney injury: a multicenter cohort study
Source: Crit Care. 2014 Sep 17;18(5):513. doi: 10.1186/s13054-014-0513-1 (PMC4189586; doi:10.1186/s13054-014-0513-1)
Supplement: Additional file 4: — Sensitivity analysis. The adjusted association between transfer status and 30-day mortality after excluding transferred patients who commenced renal replacement therapy (RRT) more than 2 days after transfer is shown. [file 13054_2014_513_MOESM4_ESM.docx]

**Additional file 4. Sensitivity analysis. The adjusted association between transfer status and 30-day mortality after excluding transferred patients who commenced RRT > 2 days after transfer**

| **Variable** |  | **Univariate OR (95% CI)** | **Multivariable OR (95% CI)** |
| --- | --- | --- | --- |
| Group | Non-transfer | 1.00 | 1.00 |
|  | Transferred and started on RRT ≤2days | 0.72 (0.43-1.20)  p=0.20 | 0.68 (0.36-1.28)  p=0.23 |
